# Comparisons of interdisciplinary ballast water treatment systems and operational experiences from ships

**DOI:** 10.1186/s40064-016-1916-z

**Published:** 2016-02-29

**Authors:** Goran Bakalar

**Affiliations:** University of Zadar, Zadar, Croatia; International Maritime Technology Consultancy, Split, Croatia

**Keywords:** Ballast water treatment, Multi-criteria analysis, Operational experience, Comparison

## Abstract

There are high functioning and low functioning ballast water treatment systems on board ships. In this study, five systems were analysed so as to methodically examine the operational difficulties for ship crew members while giving important consideration to sustainable environment practices. Multi-criteria analysis, a questionnaire, survey and interviews were used as the research method so as to ascertain and corroborate existing problems on board ships, and the reliability of the systems was calculated. The co-insistency, maintenance and the efficiency of the systems, were shown as being the major problem as there are no systems for tracking ship ballast operations from land. The treatment system that used oxidants was, through multi criteria analysis, evaluated as being the best and was ranked first. However, the survey results showed that the ship’s crew had serious problems with this system which difficult to solve during the ship’s operations with cargo. The deoxygenation system was the most appropriate according to ballast water treatment criteria in the port or at sea. The treatment system which used electrolysis with oxidant was better in terms of efficacy and the treatment system electrolysis with ultra violet light was better in terms of the criterion environment pollution footprint. During further research, it was shown that 7 % of the surveyed crew members had major problems with operating ballast water treatment systems, including the system which was ranked first through multi criteria analysis. They by-passed these systems while continuing to ballast or de-ballast. It was calculated that of the total time needed for the ballast water treatment system operation, 9 % of this time was used for repairs or maintenance of the systems. Some examples are changing a used UV bulb, cleaning the filter or controlling the amount of oxidant which would be discharged into the sea. A conclusion was made and solution was suggested. The study results emphasised taking action in the interest of protecting the natural world, with particular attention being given to environmental protection to support human life.

## Background

When large ships offload their cargo, they flood ballast tanks with seawater for stability on the return trip. Ballast water is widely used on ships to ensure manoeuvrability and stability when cargoes are unloaded (La Carbona et al. 2010). Without proper ballast, the ship’s centre of gravity is too high and can result in their capsizing. At the cargo loading port, the ballast water is pumped into the harbour in order to load more cargo. When ballast water is pumped overboard it may carry with it aquatic nuisance species that can seriously harm indigenous species in ports and coastal waters. These nuisance species may include unwanted bacteria such as *Escherichia coli* or *Vibrio cholerae* and animals such as zebra mussels. In addition, the ballast tanks are often contaminated with invasive species of animals, plants, and bacteria from the previous port. The BWMC (*Ballast Water Management Convention*) 2004 protects the sea environment through regulations regarding future ballast water treatment. Regulations and standards established by the IMO (*International Maritime Organization*) require treatment limits to be met independently and calls for vessels to carry out monitoring themselves (Albert et al. 2013). Ballast water will have to be treated once this convention comes into effect (Gregg et al. 2009).

Many technologies have been developed for ballast water treatment and fall into two broad categories: in-line and in-tank. In-line systems operate so that water is pumped from the sea to the ballast tanks routed through a processing system which kills organisms before they reach the ballast tank. In-tank systems kill the organisms after the ballast tanks are filled with seawater from the port. These are reasonably considered batch process systems. These systems treat water in the ballast tanks rather than during intake or discharge. Treatment of the ballast water can occur en route between ballast water operations and this is the principal advantage of in-tank systems. Different technologies have been suggested for ballast water treatment, such as disinfection with chlorine (Simpson 2001), injecting chemicals (La Carbona et al. 2010), adding biocides to ballast water for the neutralisation of harmful microorganisms (Chelossi and Faimali 2006), sterilisation with ozone (Perrins et al. 2006), filtration with UV (ultraviolet) light (Sutherland et al. 2013), exposure to electrochemical charge or electro-ionization (Aliotta et al. 2001), exposure to heat (Mountfort et al. 2001), and sonication (Gavand et al. 2007). Some suggested systems use acids, while others sterilisation with hydrogen peroxide (Smit et al. 2008). The deoxygenation system (Browning 2001) kills organisms in the ballast tanks by creating a water environment (inert gas + CO_2_) which is low in oxygen, higher than normal in CO_2_, and with a lower than normal seawater pH. Some systems employ multiple methods to increase effectiveness. The 2 × filtration + peracetic acid system utilises the method of passing the ballast water through the filters and adding peracetic acid. The filtration + electrolysis + ultraviolet light system provides the method of killing plants, bacteria and animals passing the ballast water through the filter and exposing them to electrolysis and ultraviolet light (Sutherland et al. 2013). The electrolysis system conducts sterilisation passing the ballast water through the filter with electrolysis (Rigby and Taylor 2001). The filtration with electrolysis, electrochlorination and waste control system uses the electrolysis method, injecting chlorine to induct a electrochemical reaction and removing the waste depending on the water quality. For this system, current density is an important parameter effecting the production of total residual chlorine in ballast or brackish water. Low current densities can avoid the production of harmful chlorine species (Lacasa et al. 2013).

A complete listing of the companies, country of origin, type of treatment, current status of testing and comments are available (Lloyd’s 2014). Numerous BWTS (*Ballast Water Treatment Systems*) have been type approved by flag administrations to meet the requirements of the IMO ballast water convention (Lloyd’s 2014; EPA Environmental Protection Agency 2011). At the moment, many ships are already equipped with ballast water treatment systems. Not all of them have treatment systems with optimal operational performances. When shipping company owners plan to buy ballast water treatment systems, they can choose between similar treatment systems with regard to the needs of their fleet. Since there is no automatic performance control to alert port authorities, they can choose cheaper and smaller systems with lower treatment costs when considering the load rate of ballast water in m^3^/h.

The objective of this study is to assess the performance of various ballast water treatment systems from the point of view of ship crew members. Real, first-hand experience can verify the impact that individuals can have on the environment. The aim of this research is to reduce the negative impact that ship operations have on the environment. The overall aim of the study is to develop processes that will lead to complete environmental sustainability in the future.

## Methods

The materials used in this research were different ballast water treatment systems and their operational processes. Methods used in this research were multi-criteria analysis, a questionnaire, survey and reliability study.

The predominant methods of killing plants, bacteria and animals in ballast water treatment systems require passing the ballast water through filters (Matheickal et al. 2001). Filters in the systems, the maintenance of the UV generator and efficacy were imperative for this research. Efficacy means whether the system performance met the IMO guidelines of not discharging the prescribed quantities of living phytoplankton, zooplankton, microbial organisms and residue of used oxidant into the water.

### Multi-criteria analysis

Multi-criteria analysis can be defined as a decision-making model that consists of a set of solutions (variants to rank or sort by the decision-maker), a set of criteria (quantitative and qualitative, economic and ecological, using different measures) and the set value (score) of each variant for each criteria (Hajkowicz and Collins 2007). The PROMETHEE (*Preference Ranking Organization Method for Enrichment Evaluations)* method was designed to analyse criteria parameters including an alternative choice of specific criteria. In PROMETHEE I partial ranking, $\varphi^{+}$ shows how much one criterion (*a*) prefers the other criteria (*b*):

1 $$ \varvec{\varphi }^{ + } \left( \varvec{a} \right) = \left( {\frac{1}{n - 1}} \right)\sum\limits_{{\varvec{b} \ne \varvec{a}}} {\varvec{\pi}\left( {\varvec{a,b}} \right)} $$

The other option ***φ***^−^ shows the weakness of one criterion against the other criteria:

2 $$ \varvec{\varphi }^{ - } \left( \varvec{a} \right) = \left( {\frac{1}{n - 1}} \right)\sum\limits_{{\varvec{b} \ne \varvec{a}}} {\varvec{\pi}\left( {\varvec{b,a}} \right)} $$

“***a***” would prefer “***b***” only if:

3 $$ \varvec{\varphi }^{ + } \left( \varvec{a} \right) \ge \varvec{\varphi }^{ + } \left( \varvec{b} \right) $$

and also:

4 $$ \varvec{\varphi }^{ - } \left( \varvec{a} \right) \le \cdots \varvec{\varphi }^{ - } \left( \varvec{b} \right) $$

In PROMETHEE II complete ranking, the ranking criteria flow in opposite directions because of the different views which are not touching each other (that is presenting incomparable and different criteria which uses different measures: e.g. economic, environmental, and social); this means that:

“***a***” would prefer “***b***” only if:

5 $$ \varvec{\varphi }\left( \varvec{a} \right)\text{ > } \cdots \varvec{\varphi }\left( \varvec{b} \right) $$

The PROMETHEE II method was particularly well-fitted to this research as it could make decisions from incomparable criteria. PROMETHEE II has been used with success to solve many problems (Behzadian et al. 2009). It was suggested that the PROMETHEE II method be used in this paper since this method is based on partial aggregation and possible decisions are compared to each other in pairs and ranked. It was then possible to select the best decision. Possible decisions (solutions) in this research were different ballast water treatment systems with common criteria (parameters) as alternative criteria was not set as an option. Compared to the well-established ELECTRE decision making method (also based on partial aggregation), the PROMETHEE II method was easier to use and able to get more robust results (Al-Shemmeria et al. 1997; Thaillandier and Stinckwich 2011).

### Criteria

The purpose of these analyses was to determine which ballast water treatment system had optimal performance from the ship crew members’ the point of view and as good as possible a performance from an ecological aspect. Five different effective possible decisions (alternatives) with type approvals were chosen to be ranked using the PROMETHEE II multi-criteria decision making method:

1. 2 × filtration + peracetic acid.
2. Filtration + electrolysis + UV generator.
3. Filtration + electrolysis + electrochlorination + waste control.
4. Electrolysis + oxidant.
5. Deoxygenation.

The importance of ecological protection was dealt with in three of the five criteria parameters. All criteria alternatives were analysed according to the following criteria parameters, as shown in Table 1.

**Table 1:** Parameters data (Lloyd’s 2014)

Ballast water treatment system	Environment pollution footprint 200/2000 m3/h	Efficacy	G8 or G9	Treatment in port or on the sea during the voyage	Number of methods
2 × filtration + peracetic acid	4.3/-	4	G9	Port	2
Filtration + electrolysis + UV	0.6/3	4	G9	Port	2
Filtration + electrolysis + electrochlorination + waste control	8.7/12.4	4	G9	Port	3
Electrolysis + oxidant	4.5/11	5	G9	Port	2
Deoxygenation	5/9	1	G8	Sea	2

Environmental pollution in this research refers to the introduction of contaminants into the natural environment that could cause adverse change. The ecological footprint used for this criterion parameter was ascertained by measuring the ballast water treatment system operation demand on the Earth’s ecosystems, i.e. the amount of natural resource capital used per annum. Efficacy was a testing score parameter, i.e. data collected from the test performance results of ballast water treatment systems recorded via the United States Marine Invasive Species Program (Dobroski et al. 2007). Whether a ballast water treatment system belongs to the G8 or G9 group depends on the test results. Tests were done during the certification process. The Ballast Water Management Convention (BWMC) and the *Guidelines for approval of ballast water management systems*—*G8* must be taken into consideration for the approval of a ballast water management system. If a certain ballast water technology uses an active substance, then, to comply with the Convention, it should be approved by the IMO in accordance with the *Procedure for approval of ballast water management systems that make use of Active Substances*—*G9*, adopted by the MEPC (Marine Environment Protection Committee) in session 53 (MEPC 2005; Tsolaki and Diamadopulos 2010). The degree of environmental friendliness of a particular ballast system determines whether it belongs to the G8 or G9 group parameter weighting. The G8 or G9 classification treatment systems and efficacy parameters are the most significant for ports and port ecology. The system that uses peracetic acid will arouse the attention of port staff more as this substance belongs to the G9 group. The number of treatment methods and whether treatment is done in the port or during the voyage is an important parameter from the ship crew members’ the point of view. Some systems employ multiple methods to increase effectiveness and some systems use just one method. If a certain ballast water technology uses one treatment method, then, it should be easier to maintain the system. If the system neutralizes the ballast water while the ship is in motion, crewmembers have more time to rest and to go ashore and visit the port during cargo operations. All these criteria parameters were weighted as per the research results collected in Table 1.

Weights simulation:

1. Environment pollution 20 %.
2. Efficacy 35 %.
3. Belonging to the group G8 or G9 20 %.
4. Treatment in the port or on the sea—during the voyage 15 %.
5. Number of the treatment methods 10 %.

Weighting of criteria is subjective and has a direct influence on the results of prioritizing strategy options. It is therefore critical that criteria weights are determined rationally and truthfully (Mutikanga et al. 2011). Efficacy was the most important criterion. Efficacy is the reason BWTS exist as it shows the amount of quality in terms of treating ballast water. This criterion was given a weighting of 35 % for ranking. The two next most important criteria were ecological: environment pollution and belonging to the group G8 or G9 and were given a ranking weight of 20 % each. The remaining 25 % was given to the two remaining criteria. As the most important criterion to the ship’s crew is treatment in port or at sea, it was given 15 %, while the number of methods of a particular BWTS was given the remaining 10 %.

### Survey of experiences from ships

The objective of this survey was to ascertain difficulties regarding the operational process of ballast water treatment systems. This was based on ship crew members’ operational experience and their personal opinion. The survey raises considerable doubts as to the reliability of quick answers to research questions, whether of a quantitative or qualitative nature (Charmaz 1995). The aim was to collect qualitative information to meet the objectives of the research. A simple exploratory questionnaire was created with four questions. A questionnaire is a research instrument and basic scientific method whose purpose is to gather information from respondents. The discovery of reflexive progression in interviewing is very important for research (Miller and Glassner 1997). This study provides survey findings from a sample of 68 experienced ship captains and bridge officers, 51 of whom attended maritime courses and 18 from the maritime crewing agency. These courses were taught in Split, Sibenik and Rijeka, Croatia, at the Maritime School, and the students were interviewed in the period from 20/12/2014 to 1/3/2015. Interviews were carried out after the survey research data was collected. An on-board experience questionnaire was done for this research and a summary of the survey results are given and explained further in this paper.

### The questionnaire

The questionnaire was made up of the following:

Circle one answer only.

1. How many years have you worked on board ships?
  - one year.
  - three years.
  - five years.
  - more than five years.
2. I work on ships as a:
  - bridge officer.
  - captain.
3. There is a ballast water treatment system installed on the ship that I work on:
  - Yes.
  - No.
4. There were some functional problems while the ballast water treatment system was in operation:
  - Yes.
  - No.

### The reliability and availability of the systems

Malfunction of the ballast water treatment systems on board ships is a criterion that has never been reported or included in any research, but could prove to be the most important factor in ballast water treatment system performance. Performance reliability of certain ballast water treatment systems on board ships have indicators which should be observed, followed and analysed over a longer period of system exploitation. Reliability is the ability of the system for operational work without interruption (Pham 2000). Predictions of potential failures caused by software or hardware errors, as well as potential failures in mechanical part performance, can only be predicted once findings from the experience in handling technologically-similar systems on board ships are found. External factors on ships are one of the most important factors, because the dynamics of movements are constantly under external influences. Likewise, operator reliability and exposure to high temperatures are also very important in assessing the reliability of the proposed system (Siewiorek and Swarz 1982).

The reliability of these types of devices can be shown as an exponential function of the time interval if the time interval is considered to be the useful lifespan of the device. For electronic systems such as sophisticated devices for the on-board ballast water treatment of ships, the failure density function has a form of an exponential distribution, so the failure frequency function is the same by definition (Turban et al. 2003):

6 $$ \lambda \left( t \right) = \frac{f\left( t \right)}{R\left( t \right)} = \frac{{\lambda e^{ - \lambda t} }}{{e^{ - \lambda t} }} = \lambda $$

In the Eq. (6), we can see that the failure frequency function has a constant value (*λ*). As such, the equation for the reliability function *R*(*t*) (exponential law of reliability) has the form of:

7 $$ R\left( t \right) = e^{ - \lambda t} $$

The computing subsystem of some of the ballast water treatment systems on board ships can be found in three states: proper function, procedural failure and non-procedural failure (Shooman 2002). Computing subsystem reliability depends on the probability of proper function and failures in a specified time. The probability of a computing subsystem of certain ballast water treatment systems on board ships going from a state of proper function to a state of non-procedural failure is:

8 $$ P = \left( {t + \varDelta t} \right) = \lambda \varDelta tP_{c} \left( {1 - F} \right) + \left( {1 - \mu \varDelta t} \right)P_{i.o} \left( t \right) $$

where $\varvec{P}_{{\varvec{i}.\varvec{o}}}$ probability of non-procedural state, $\varvec{P}_{\varvec{c}}$ probability of proper functioning state, ***μ*** frequency of repairs, ***F*** redundancy failure detection ratio, ***Δt*** time lag/time interval, ***λ*** malfunction index.

*The Malfunction index* is the relation between malfunctioning components and operational components:

9 $$ \lambda = \frac{1}{{P_{c}  }} \frac{{d P_{f} }}{\varDelta t } $$

where *P*_*c*_ is the number of components which remained operational after a specified time, and *P*_*f*_ is the number of components which malfunctioned after the specified time of functioning.

Redundancy is a characteristic of the quality of the computing system that ensures failure avoidance when one part of the system fails. This is generally ensured through the use of additional software, through the reliability of the two redundant systems working in parallel and with a known malfunction index for these types of devices in predefined ship conditions.

It can be assumed that the computing part of the subsystem in the system with two parallel subsystems, one of which is redundant, will be regularly maintained by the operator and through the self-diagnosis function for error removal. In this way, the possibility of malfunction of some ballast water treatment systems on board ships is reduced.

## Results and discussion

### Multi-criteria analysis of the systems

**Fig. 1:**
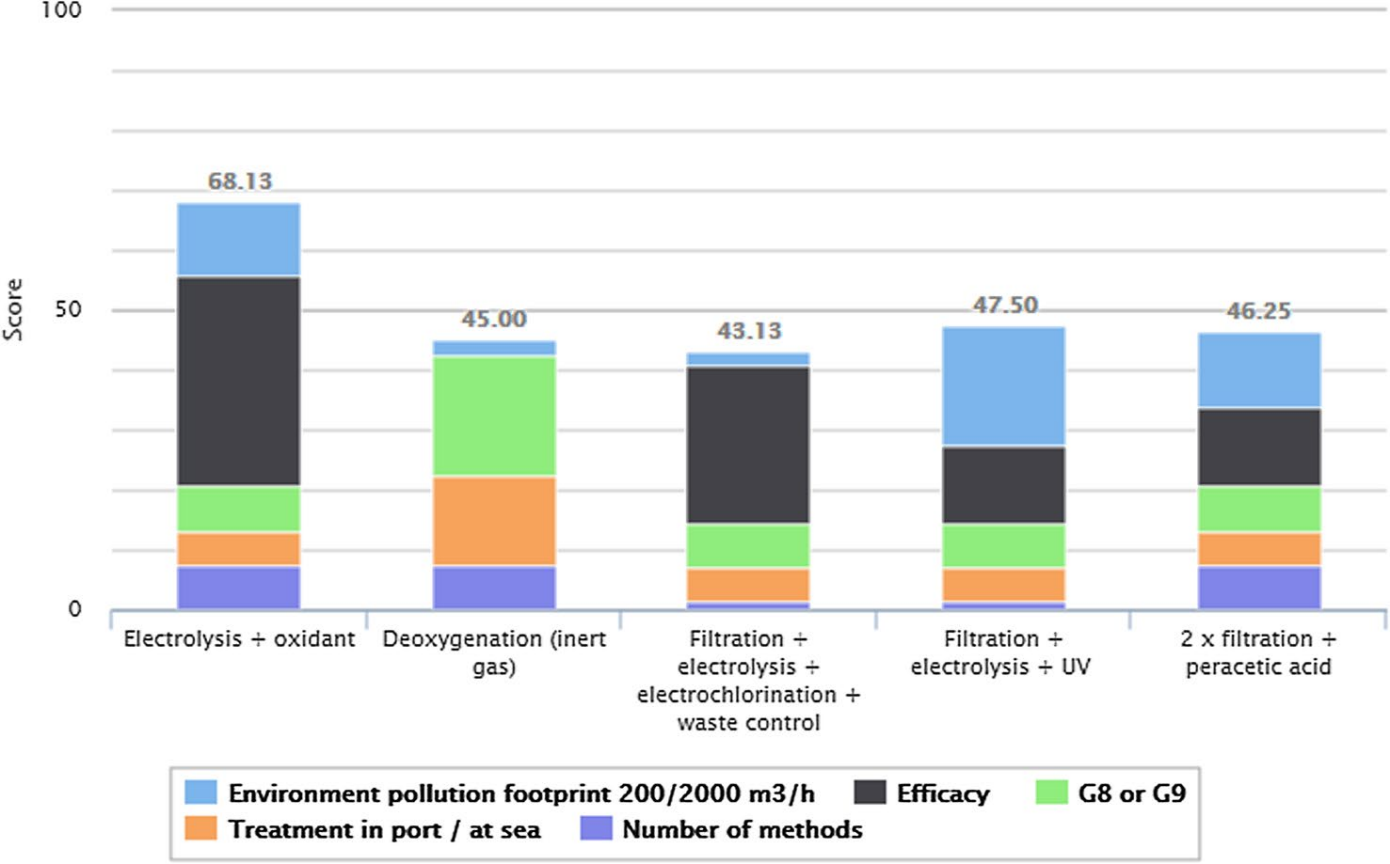
Multi-criteria analysis PROMETHEE II results

The D-Sight computerised visual method or visual projection of PROMETHEE II analysis, as seen in Fig. 1, has projected and showed the most appropriate system in ranking the results.

The criteria parameters contribution to the solutions is shown in five different colours on the figure. The treatment system of electrolysis + oxidant technology was highly successful in this computational analysis with a percentile of 68.1 %. The value of the criteria parameter environmental pollution for the BWTS filtration with electrolysis and UV, was the most important criterion for ranking second with a percentile of 47.5 %. The system 2 × filtration with peracetic acid technology was ranked third with a percentile of 46.25 %. Crucial to its coming in third place was the high quality of its efficacy value, as well as environmental pollution, in terms of criteria parameters. As one of the analysis criteria was the treatment of ballast water in the port or at sea, the deoxygenation system received a percentile of 45 % as it is the only ballast water treatment system which can be used while at sea. The system which was ranked last was the filtration + electrolysis + electrochlorination + waste control system. It can be confirmed that this system has a high value in terms of the efficacy criteria parameter. However, because of its high environmental pollution footprint, as well as the highest number of treatment methods, received a percentile of 43.1 % and was ranked fifth in this multi criteria analysis.

### Results of the survey

Three students from the courses (out of the 51 surveyed) responded to questions 3, 4 and 5 of the questionnaire affirmatively. In the maritime crewing agency, two experienced officers responded affirmatively to questions 3, 4 and 5. Of the 5 ship captains who were surveyed and during the interview confirmed that they had problems with the operating of the BWTS, one of the surveyed captains worked on a ship with an installed BWTS using the treatment method of electrolysis and oxidant.

The problem that this chief officer referred to in regards to treatment system that uses electrolysis and oxidants was TRO (total residue oxidant). The measured TRO can provide an indication of treatment performance but not a direct measure of discharge standards. TRO was a problem for the TRO sensor unit in the deballastation process. Sensors would inhibit the operation of the ballast water treatment system due to the TRO value being too high. As the TRO was not at the required level, the residual TRO level of the treated ballast water was over 0.2 mg/L for the entire time, caused by an improperly dosed neutralizer in the neutralization unit of the system. The additional substance was most probably insufficient to automatically neutralize residual oxidants instantly and the system kept shutting down according to the interviewed chief officer. During the interview, two bridge officers that were surveyed said they were on a ship with an installed system that uses filtration, electrolysis and UV light. They said that it can take from three to three and a half hours to replace a UV bulb in a BWTS with a UV generator. Bulbs burn out often and in the case of a bulb burning out a second time, they by-passed the UV ballast water treatment system due to lack of time for cargo operations. According to another bridge officer who was interviewed for the survey, filters in the BWTS that uses filtration with UV light were a constant problem for one particular ship in a Brazilian port. The system was not able to operate due to filter problems. This problem was also referred to by another bridge officer who was on the ship with installed BWTS that uses filtration and UV light. All of officers interviewed stated that they by-passed the treatment system and continued the ballasting or de-ballasting process to avoid shutdowns. No reports were done at the time. The complete survey results can be found in Table 2. Ballast water treatment systems are not always in optimal operational condition and ships’ crew members by-pass them, as often occured with automatic oil discharge content monitors in the past (McLaughlin et al. 2014; Bakalar 2014). This is an unacceptable facet of the ballast water treatment systems’ implementation. Research regarding this problem should continue in the future so as to observe how survey results vary over time. Trend analysis reports would allow the survey response data to be charted over time and for it to be published in scientific journals.

**Table 2:** Survey results

	BWTS on board	No BWTS on board	Operational problems	Total sample surveyed	Total % with operational problems (%)
Surveyed Crew members attending courses	5	46	3	51	5.9
Surveyed Crew members in the agency for employment	4	14	2	18	11.1
Total	9	60	5	69	7.3

### The reliability and availability of the systems

The malfunction index or maintenance failure which cannot be diagnosed and automatically eliminated for these types of devices ranges from *λ* = 0.5 to *λ* = 0.2 (Bakalar 2013; Lovric 1989). *λ* can only be determined from on board ship operational history which is at present unavailable for ballast water treatment systems. The mean value of the malfunction index was the most optional value in this calculation and it was taken for all systems in common. If the mean is taken as being *λ* = 0.35 for each of the serially attached subsystems and this value is included in the equation of reliability for 1000 h of work, we can obtain a reliability value for each of the two computing systems individually, where one is redundant:

10 $$ R\left( t \right) = e^{ - \lambda t} = e^{ - 0.35} = 0.704688 $$

The total reliability of the whole computing subsystem as part of the ballast water treatment system on board ships which consists of two units, one of which is software redundant, is:

11 $$ R\left( t \right) = R_{u} \left( {1 - \lambda T} \right) = 0.916097 $$

As such, the reliability of this subsystem has been proven according to the analysis of the results of the reliability of the computing system, which is a subsystem of a ballast water treatment system on board ships. The computing subsystem reliability with the possible use of the redundant software is 0.916 or 91.6 %.

## Conclusions

This paper reviews ballast water treatment systems in three parts regarding data and methods: multi-criteria analysis, survey on real experience, reliability and availability study. According to the multi-criteria analysis results, the electrolysis + oxidant technology treatment system had a high success rate of 68.1 %. Many aspects were relevant and important. The electrolysis management system that uses oxidants was the most appropriate system from an ecological standpoint, while determining the quality of the treated ballast water was the most acceptable from an environmental point of view. The multi-criteria analysis parameters and scenarios did not consider the functional and operational problems of the systems. It can be clearly concluded from the survey result of 7.3 % from all participants and interviews that ship officers by-pass ballast water treatment systems due to lack of time for rectifying malfunctions, failures, maintenance or spare parts replacement in ballast water treatment systems. The survey results indicate that ballast water treatment systems can become faulty and shut down when operation sensors indicate improper function of the sub-systems. Even the system which utilises oxidant, which was successful in the multi criteria analysis, was shown to have many problems regarding controlling oxidants. This implies an unclear future for port environment protection management, where the ballast water is pumped into the harbour in order to load cargo. The research in this paper aids in understanding how important it is to learn more about ballast water treatment system experiences from crewmembers, since the survey of operational experiences from ships has proven that more time would be needed for the maintenance of ballast water management systems. The total calculated reliability of the entire computing subsystem as part of a particular ballast water treatment system on board ships which consists of the two units of which one is software redundant, was 0.916 or 91.6 %. This means that for 8.4 % of the operational time, any of the mentioned systems could be in failure or under repair. This is a significant risk for the operation of ballast water treatment systems. The results of this research are a warning to relevant stakeholders in the maritime industry. If the system does not operate well, or not at all, the price of treatment is higher, as is air and water pollution, and the active substances used, e.g. oxidant residue, become unmanageable and out of control. Environmental hazards need to be managed. One possibility would be a mechanism to monitor ballast water systems operation continually on board ships. A check-up system should be close as possible to real time. Sensors should report malfunctions to the port authorities immediately after any questionable or doubtful performance of ballast water treatment system occur.
